# Supplementary material for: Longitudinal study of risk factors predicting cannabis use disorder in UK young adults and adolescents
Source: Commun Med (Lond). 2025 Jul 19;5:300. doi: 10.1038/s43856-025-01018-y (PMC12276291; doi:10.1038/s43856-025-01018-y)
Supplement: Supplementary file 2 — Supplementary information [file 43856_2025_1018_MOESM2_ESM.pdf]

SUPPLEMENTARY INFORMATION

Title: Longitudinal study of risk factors predicting cannabis use disorder in UK young adults and adolescents

Authors: Martine Skumlien[1,2], Darcy Jones[1], Claire Mokrysz[3], Rachel Lees[1], Kat Petrilli[2], Shelan Ofori[3], Will Lawn[4], H Valerie Curran[3], Tom P Freeman[1]

Affiliations:

[1]Addiction and Mental Health Group (AIM), Department of Psychology, University of Bath, Bath, UK

[2]Department of Addictions, King's College London, London, UK

[3]Clinical Psychopharmacology Unit, University College London, London, UK

[4]Department of Psychology, King's College London, London, UK

Address correspondence to: Martine Skumlien, ms4068@bath.ac.uk

Supplementary Table 1. Results of ordinal logistic regressions predicting CUD Level (no, mild, moderate, severe) by baseline factors in n=117 participants who used cannabis 1-7 days per week at baseline, controlling for age of first cannabis use.

	B	Standard error	*p*	Odds ratio	95% CI, lower	95% CI, upper
Female	-0.21	0.36	.55	0.81	0.40	1.64
Male	Reference group					
Adolescent	1.57	0.48	<.001	4.81	1.89	12.25
Young adult	Reference group					
CUD	2.31	0.59	<.001	10.04	3.18	31.74
No CUD	Reference group					
After COVID-19 lockdown	-0.43	0.39	.27	0.65	0.30	1.40
Before COVID-19 lockdown	Reference group					
Days/week cannabis use	0.03	0.11	.77	1.03	0.84	1.27
Age of first cannabis use	0.07	0.09	.42	1.07	0.90	1.28
AUDIT score	0.02	0.04	.66	1.02	0.94	1.10
HSI score	-0.01	0.31	.99	1.00	0.54	1.82
Negative life events	0.04	0.11	.70	1.04	0.85	1.28

Abbreviations: AUDIT, Alcohol Use Disorder Identification Test; COVID, coronavirus disease; CUD, cannabis use disorder; HSI, Heaviness of Smoking Index.
