# Supplementary material for: Longitudinal study of risk factors predicting cannabis use disorder in UK young adults and adolescents
Source: Commun Med (Lond). 2025 Jul 19;5:300. doi: 10.1038/s43856-025-01018-y (PMC12276291; doi:10.1038/s43856-025-01018-y)
Supplement: Supplementary file 3 — Reporting Summary [file 43856_2025_1018_MOESM3_ESM.pdf]

nature portfolio

Corresponding author(s): Martine Skumlien

Last updated by author(s): Jun 17, 2025

Reporting Summary

Nature Portfolio wishes to improve the reproducibility of the work that we publish. This form provides structure for consistency and transparency in reporting. For further information on Nature Portfolio policies, see our Editorial Policies and the Editorial Policy Checklist.

Statistics

For all statistical analyses, confirm that the following items are present in the figure legend, table legend, main text, or Methods section.

n/a	Confirmed	
☐	☒	The exact sample size (n) for each experimental group/condition, given as a discrete number and unit of measurement
☐	☒	A statement on whether measurements were taken from distinct samples or whether the same sample was measured repeatedly
☐	☒	The statistical test(s) used AND whether they are one- or two-sided<br>*Only common tests should be described solely by name; describe more complex techniques in the Methods section.*
☐	☒	A description of all covariates tested
☐	☒	A description of any assumptions or corrections, such as tests of normality and adjustment for multiple comparisons
☐	☒	A full description of the statistical parameters including central tendency (e.g. means) or other basic estimates (e.g. regression coefficient) AND variation (e.g. standard deviation) or associated estimates of uncertainty (e.g. confidence intervals)
☐	☒	For null hypothesis testing, the test statistic (e.g. F, t, r) with confidence intervals, effect sizes, degrees of freedom and P value noted<br>*Give P values as exact values whenever suitable.*
☒	☐	For Bayesian analysis, information on the choice of priors and Markov chain Monte Carlo settings
☒	☐	For hierarchical and complex designs, identification of the appropriate level for tests and full reporting of outcomes
☐	☒	Estimates of effect sizes (e.g. Cohen's d, Pearson's r), indicating how they were calculated

Our web collection on statistics for biologists contains articles on many of the points above.

Software and code

Policy information about availability of computer code

Data collection	No software was used to collect data for this study
Data analysis	Data were analysed with IBM SPSS Statistics version 29.0.2.0

For manuscripts utilizing custom algorithms or software that are central to the research but not yet described in published literature, software must be made available to editors and reviewers. We strongly encourage code deposition in a community repository (e.g. GitHub). See the Nature Portfolio guidelines for submitting code & software for further information.

Data

Policy information about availability of data

All manuscripts must include a data availability statement. This statement should provide the following information, where applicable:

- Accession codes, unique identifiers, or web links for publicly available datasets
- A description of any restrictions on data availability
- For clinical datasets or third party data, please ensure that the statement adheres to our policy

The participants of this study did not give written consent for their data to be shared publicly and the data supporting this research are therefore not available.

Research involving human participants, their data, or biological material

Policy information about studies with human participants or human data. See also policy information about sex, gender (identity/presentation), and sexual orientation and race, ethnicity and racism.

Reporting on sex and gender	Gender was self-reported by participants from the options 'male', 'female', 'other', and 'prefer not to say'. Gender was not considered in our statistical analyses but groups were recruited to contain 50:50 males:females. In this study, 120 participants identified as female and 112 identified as male. None identified as 'other' or indicated 'prefer not to say'.
Reporting on race, ethnicity, or other socially relevant groupings	Participants self-identified their race/ethnicity from the following categories based on UK census data: White - 1. English/ Welsh/Scottish/Northern Iris/British, 2. Irish, 3. Gypsy or Irish Traveller, 4. Any other white background, please describe; Mixed/Multiple ethnic groups - 5. White and Black Caribbean, 6. White and Black African, 7. White and Asian, 8. Any other Mixed/Multiple ethnic background, please describe; Asian/Asian British - 9. Indian, 10. Pakistani, 11. Bangladeshi, 12. Chinese, 13. Any other Asian background, please describe; Black/African/Caribbean/Black British - 14. African, 15. Caribbean, 16. Any other Black/African/Caribbean background, please describe; Other ethnic group - 17. Arab, 18. Any other ethnic group, please describe. For simplicity, these were collapsed into the following categories for this manuscript: White (n=149), Mixed (n=29), Asian (n=32), Black (n=12), Other (n=8), prefer not to say (n=2). Our sample roughly reflects the racial/ethnic makeup of London.
Population characteristics	Participants were purposively recruited into four groups based on their age and cannabis use at baseline: adolescent people who use cannabis (PWUC, n=61), young adult PWUC (n=56), adolescent controls (n=57), and young adult controls (n=58). Adolescents were 16-17 years and adults were 26-29 years.
Recruitment	Participants were recruited from the Greater London area via school assemblies, physical posters and flyers, and social media advertisements. The CannTeen study used purposive sampling to recruit balanced groups of PWUC and controls, and within these groups, equal numbers of young adults and adolescents and males and females. While this was necessary to ensure a sufficient number of people who used cannabis frequently in both age and gender groups, it inevitably reduced the representativeness of our sample.
Ethics oversight	University College London ethics committee, project ID 5929/003.

Note that full information on the approval of the study protocol must also be provided in the manuscript.

Field-specific reporting

Please select the one below that is the best fit for your research. If you are not sure, read the appropriate sections before making your selection.

☐ Life sciences ☒ Behavioural & social sciences ☐ Ecological, evolutionary & environmental sciences

For a reference copy of the document with all sections, see nature.com/documents/nr-reporting-summary-flat.pdf

Behavioural & social sciences study design

All studies must disclose on these points even when the disclosure is negative.

Study description	This is a longitudinal observational study using quantitative data from the baseline and 12-month follow-up sessions of the CannTeen study.
Research sample	The overall aim of the CannTeen study was to explore whether associations between cannabis use and multiple psychological, cognitive, and biological factors differed between adolescents and young adults. Therefore, participants were purposively recruited into four groups based on their age and cannabis use at baseline: adolescent people who use cannabis (PWUC, n=61), young adult PWUC (n=56), adolescent controls (n=57), and young adult controls (n=58) from the general Greater London population. Within each age/cannabis group, we recruited 50:50 males and females. This form of purposive recruitment meant our sample is not representative of the UK or London population. However, this was necessary to ensure a sufficient number of people who used cannabis.
Sampling strategy	Purposive and snowball sampling was used. The CannTeen study was powered to to be able to detect a cross-sectional group difference in cannabis dependence between teenage and adult cannabis users, as this is a robust finding with a quantified effect size (Chenet al., 2009, Ehlerset al., 2010, Le Stratet al., 2015). These studies of addiction consistently show an increased likelihood of adolescent cannabis dependence in comparison to adult cannabis dependence, with an odds ratio of roughly three. An odds ratio of 3 is equivalent to a Cohen's d=0.6 (https://www.psychometrica.de/effect_size.html). Therefore, in order to detect a cross-sectional difference at baseline in cannabis dependence between adolescent and adult cannabis users (with an alpha of 0.05 and a desired power of 0.95), we would need 148 cannabis users, split evenly between teenagers and adults. We in fact recruited 147 cannabis users. And we therefore recruited a similar number of controls. 232 participants were included in the current analyses. [32]. With 232 participants and a Bonferroni-corrected alpha-level of .05, we had 83.7% power to detect a small-to-medium effect size of OR=2.575 for each predictor. This is an approximation only, as the function used assumes a balanced two-group structure for the independent variable.
Data collection	All sessions taking place on or before March 23rd 2020 (i.e., before the COVID-19 lockdown) were completed in-person at University

Data collection	College London Clinical Psychopharmacology Unit. All sessions after the lockdown took place online. Paper-and-pencil materials were used. Only one researcher was typically present with participants and researchers were not explicitly blinded to hypotheses.
Timing	The first session took place on 1st November 2017 and the final session took place on 20 November 2020.
Data exclusions	The CannTeen study included 274 participants. Of these, 234 (85.4%) completed the follow-up assessment, and 232 had complete data and were included in the study. The two excluded participants had missing values on the negative life events measure
Non-participation	Please see 'data exclusions'. Reasons for drop-out were not reported.
Randomization	Not applicable, as this is an observational study.

Reporting for specific materials, systems and methods

We require information from authors about some types of materials, experimental systems and methods used in many studies. Here, indicate whether each material, system or method listed is relevant to your study. If you are not sure if a list item applies to your research, read the appropriate section before selecting a response.

Materials & experimental systems

n/a	Involved in the study	
☒	☐	Antibodies
☒	☐	Eukaryotic cell lines
☒	☐	Palaeontology and archaeology
☒	☐	Animals and other organisms
☒	☐	Clinical data
☒	☐	Dual use research of concern
☒	☐	Plants

Methods

n/a	Involved in the study	
☒	☐	ChIP-seq
☒	☐	Flow cytometry
☒	☐	MRI-based neuroimaging

Plants

Seed stocks	*Report on the source of all seed stocks or other plant material used. If applicable, state the seed stock centre and catalogue number. If plant specimens were collected from the field, describe the collection location, date and sampling procedures.*
Novel plant genotypes	*Describe the methods by which all novel plant genotypes were produced. This includes those generated by transgenic approaches, gene editing, chemical/radiation-based mutagenesis and hybridization. For transgenic lines, describe the transformation method, the number of independent lines analyzed and the generation upon which experiments were performed. For gene-edited lines, describe the editor used, the endogenous sequence targeted for editing, the targeting guide RNA sequence (if applicable) and how the editor was applied.*
Authentication	*Describe any authentication procedures for each seed stock used or novel genotype generated. Describe any experiments used to assess the effect of a mutation and, where applicable, how potential secondary effects (e.g. second site T-DNA insertions, mosiacism, off-target gene editing) were examined.*
